# Protective and Risk Factors for Suicidal Behaviour in Self-Declared LGBTIQ+ Adolescents

**DOI:** 10.3390/bs14050422

**Published:** 2024-05-18

**Authors:** David Sánchez-Teruel, Francisca López-Torrecillas, María Auxiliadora Robles-Bello, Nieves Valencia-Naranjo

**Affiliations:** 1 Department of Personality, Assessment and Psychological Treatment, Faculty of Psychology, University of Granada, 18012 Granada, Spain; dsteruel@ugr.es (D.S.-T.); fcalopez@ugr.es (F.L.-T.); 2 Spanish Society of Suicidology, 28015 Madrid, Spain; 3 Psychology Department, University of Jaén, 23071 Jaen, Spain; nnaranjo@ujaen.es

**Keywords:** suicidal behaviour, adolescents, LGBTIQ+, entrapment, resilience

## Abstract

Background: Adolescents who identify as sexual minorities often face social stigmatisation, which can lead to increased adversity and a higher risk of suicidal behaviours. However, there are also protective factors that may promote resilience to these risk behaviours. This study aims to identify factors that predict resilience in sexual minority adolescents with high suicidal vulnerability. Methods: The study sample comprised 78 self-reported LGBTIQ+ adolescents aged between 13 and 18 years old (M = 16.11, SD = 1.56) who had previously attempted suicide. They completed several psychosocial instruments to measure risk and protective variables related to suicidal vulnerability. Results: Entrapment was found to be the most predictive risk variable for suicide attempts. However, protective variables promoting resilience were also found, such as adequate parental communication, life satisfaction, and cognitive reframing. Discussion: The findings related to prevention of suicide attempts are discussed and we highlight the urgent need to enhance certain internal and contextual protective factors to promote resilience in the face of suicidal vulnerability in certain clinical subpopulations exposed to highly adverse situations.

## 1. Introduction

In recent years, the risk of suicide in self-identified lesbian, gay, bisexual, transgender, intersex, queer, and other sexual minorities (henceforth LGBTIQ+) [1] has been the subject of increased scientific concern because of its strong association with suicidal behaviour [2]. The rate of suicide in LGBTIQ+ people in most countries is unknown, but identifying with a sexual identity or orientation other than heteronormative is generally estimated to be associated with an increased risk of attempted suicide, especially in adolescents and young adults [3,4]; however, it is people who self-identify as transgender who report a higher prevalence of suicide attempts [5].

The scientific literature provides information on multiple risk factors associated with self-harm in adolescents and young people in the general population [6,7]. However, few studies have assessed risk factors (social isolation, low self-esteem and substance abuse, depression, anxiety or stress) related to suicidal behaviours in self-reported LGBTIQ+ adolescents [3,8,9]. It has been suggested that these adolescents and young people may conclude that they do not have viable solutions to deal with some of the social adversities they are exposed to because of their sexual identity or orientation, so they develop a feeling of “entrapment” or helplessness [10]. In 69 countries, non-heteronormative identity or orientation is criminalised, perceiving the difference as a tremendous burden [1,3]. These adversities can lead to stigmatisation and discrimination [11], as well as experiences of family rejection, bullying or cyberbullying, intimidation, and abuse [3], especially in the absence of early adult support [12]. The result may be to consider suicidal ideation as a viable alternative that may lead to suicide attempts [13]. Such thinking can be interrupted when protective factors are available; namely, by minimising the risk or limiting the person’s conclusion that there are no solutions to adverse situations [14]. Focusing exclusively on the study of risk factors is limiting [15,16], offers a biased view of reality, and does not provide the necessary tools to create effective suicide prevention strategies [17]. Thus, current approaches suggest addressing both risk and protective factors that may promote greater resilience to suicide [18]. This is because there are many people who present risk factors that promote high vulnerability but who do not engage in suicidal behaviour and exhibit a high level of resilience to this and associated behaviours [16,19,20,21].

Suicide resilience includes a set of skills, resources, and competences that enable the regulation of thoughts, feelings, and attitudes towards suicide [22]. Protective factors may include personal characteristics (e.g., positive beliefs and feelings about oneself and satisfaction with one’s life); external resources (e.g., beliefs and feelings that help is available in complex situations or in the face of suicidal ideation); and achievement of emotional stability (e.g., positive beliefs about the ability to regulate negative emotions in situations that act as stressors). Several protective factors that promote resilience to suicidal behaviour have been observed in LGTBIQ+ adolescents and young people [23].

In this regard, social support is a modulating variable that has a positive effect on emotional well-being and mental health in these groups [24]. Toro et al. [25] argue that people belonging to this group need strong social support to foster personal identity, and, in addition, this identity helps to achieve both short- and long-term goals, whether personal or social. Social support decreases the reactivity of prejudice in society [26] (Hall, 2018), i.e., having supportive people to count on helps LGTBIQ+ people ensure that existing prejudices do not have a negative impact on their lives and do not produce negative consequences or psychopathology [27]. Other studies have also noted that social support, whether from friends, family, school, or community, plays an essential role in the development of resilience in LGTBIQ+ people [28,29]. Within social support, family support is particularly important, and certain behaviours that foster affection and communication in families of sexual minority youth have been found to be a strong predictor of psychological and emotional well-being and resilience, as well as a protective factor for mental distress [30]. However, it appears that many of these protective variables have not been measured in the LGTBIQ+ diverse population, let alone assessed in relation to resilience. Furthermore, this type of protective variable has never been related to a variable that has been reported to be equally protective, such as reappraisal [23,31], just as there have been few approaches to studying the variable emotional self-regulation together with suicidal behaviour in this group [32], despite it appearing to be an important factor in suicidal behaviour in the general adolescent population [33].

Therefore, it is conceivable that demographic and psychosocial factors that may influence suicidal vulnerability and resilience to suicide attempts in LGBTIQ+ adolescents and young people are still unknown. Consequently, the aim of our study is to determine the factors that can predict risk and resilience in these adolescents with previous suicide attempts. We might hypothesise not only risk variables associated with suicidal behaviour—associated with anxiety and depression, negative experiences in the school and family context—that may vary depending on the type of sexual orientation; there may also be protective variables that will predict a resilient outcome to suicidal behaviour in this group of people—good levels of reported psychosocial well-being, adequate communication in the family environment, good emotional regulation, and positive levels of cognitive reappraisal in the face of life adversities at this stage of development.

## 2. Materials and Methods

### 2.1. Participants

The initial sample consisted of 162 adolescents with the following inclusion criteria: (1) Aged between 10 and 19 years old (according to the conceptualisation of adolescence by the World Health Organisation [34]); (2) Residing in Spain; (3) Self-declared as LGBTIQ+; (4) One or more previous suicide attempts (last year) (Item 4 of the Ask Suicide-Screening Questions-ASQ short scale by Horowitz et al. [35]); (5) Subjects and parents or guardians have read the study information sheet and signed their informed consent. (6) Parents or guardians had to provide an email address along with their child’s initials. The final sample consisted of 78 adolescents aged 13–18 (M = 16.11; SD = 1.56), 41–52.6% self-reported as female, 33–42.3% as male, and 4–5.1% did not identify their gender (Table 1). All participants and at least one parent or guardian gave consent. The study was reviewed and approved by the Research Ethics Committee of a public university in southern Spain (Code: ABR.20/4.PRY) and all protocols outlined in the 1964 Helsinki declaration and its subsequent amendments were followed [36].

### 2.2. Measures

A demographic questionnaire was administered to participants, which collected information about age, gender, socio-economic status, educational attainment, stressful situations experienced in the past year, triggering situations, and the number of previous suicide attempts.

The Emotion Regulation Questionnaire (ERQ from Gross and John [38], adapted to the Spanish population by Cabello et al. [39]). This test provides a ten-item assessment of emotion regulation, rated on a scale from 1 (strongly disagree) to 7 (strongly agree). It has two subdimensions: cognitive reappraisal, which involves modifying emotional reactions to alter the emotional experience, and expressive suppression, which involves evaluating changes in emotional expression and hiding emotions without modifying them. The Spanish version of the scale has a comparable structure to the original and demonstrates adequate psychometric properties, as reported by Cabello et al. [39].

The Reappraisal Index (RI), developed by Fritz [31], was adapted for a Spanish population with homosexual orientation by Lorabi et al. [23] to differentiate between individuals who can positively re-evaluate stressful events and those who cannot. The RI measures a person’s capacity to shift one’s perspective on a challenging situation towards a more positive or less negative perception of it. Lorabi et al. found that a Likert-type response scale with nine items ranging from ‘1 = never/not at all’ to ‘5 = always/many times’ exhibited satisfactory psychometric properties in the Spanish homosexual population.

The Lubben Social Network Scale-6 (LSNS-6) is a reduced version of the original scale developed by Lubben [40] and the short version by Lubben et al. [41], measuring perceived social support. It was translated and adapted to the Spanish population by Fernández-Ballesteros, Reig, and Zamarrón [42]. The scale consists of six items, each with six response options ranging from ‘0 = none’ to ‘5 = nine or more people’. The Spanish version of the scale demonstrated adequate psychometric properties [42]. Other studies in Spain have also demonstrated its methodological suitability for measuring social support in the aftermath of adverse situations [43].

The Psychological Well-being Scale (PWS) was developed by [44] Sánchez-Cánovas. The complete scale consists of sixty-five items divided into four subscales, but only the subjective well-being subscale was used in the present study. This subscale measures satisfaction with life, as well as positive and negative affect. It consists of ten Likert-type items with values ranging from 1 (never or almost never) to 5 (always).

The Scale for Evaluating Parental Educational Style Towards Adolescents-SEPES, from Oliva, Parra, Sánchez-Queija, and López [45], consists of forty-one items. Respondents rate each item on a Likert scale ranging from 1 (completely disagree) to 6 (completely agree). The items are grouped into six dimensions: affection and communication, promotion of autonomy, behavioural control, psychological control, disclosure, and humour. In this study, we only used the affect and communication sub-dimension, consisting of eight items that measure the expression of support and affection from parents, their availability, and the fluency of communication.

The Entrapment Scale Short-Form (E-SF) by [46] De Beurs et al. was adapted to Spanish by Ordóñez-Carrasco et al. [47] to measure entrapment in individuals at high risk of suicide. Entrapment is defined as the attempt to escape from unbearable situations or negative thoughts that cause great suffering. The scale is based on the original sixteen-item scale by Gilbert and Allan [48] and consists of four items that are answered using a five-point format (0 = ‘not at all like me’, 4 = ‘extremely like me’). The reliability of the extended version in the Spanish samples was 0.97, according to Ordóñez-Carrasco et al. [47].

The Depression, Anxiety and Stress Scale (DASS-21) by [49] Antony et al. was adapted for a sample of the Spanish-speaking clinical population by Daza et al. [50]. It consists of twenty-one items—spread across 3 subscales, each with 7 items—using a Likert-type format with 4 categories (ranging from 0 = ‘not applicable to me at all’ to 3 = ‘applies to me very much or most of the time’). The assessment measures depression by evaluating aspects related to low positive affect such as dysphoria, hopelessness, sadness, or anhedonia. Anxiety is evaluated by assessing aspects related to psychophysiological activation or autonomic arousal, and stress measures difficulty in relaxation, nervous excitement, agitation, irritability, and impatience. The reliability level was similar to the English version.

The Suicidal Behaviour Assessment Scale (SENTIA brief) by Díez-Gómez et al. [51] measures risk factor-based suicidal vulnerability in Spanish adolescents using five dichotomous (yes/no) items. It has three subdimensions: suicidal act/planning (5, 3), suicidal communication (4), and suicidal ideation (1, 2). In this study, only the first four items were used to assess suicidal vulnerability. In its original version, this measure showed no gender variability, and its reliability was high (omega = 0.97).

The Resilience Scale for Suicide Attempts (SRSA-18 Adolescents and Youth) by Sánchez-Teruel et al. [15] measures resilience to suicide and predicts future suicide attempts. The scale consists of eighteen items, classified into three sub-dimensions: internal protection, emotional stability, and external protection, with responses given on a Likert-type scale (ranging from 0—never to 4—always). It presents high correlations with other scales measuring resilience in stressful situations [52,53] (14-item Resilience Scale-RS-14 and CD-RISC-25-Connor and Davidson Resilience Scale) and resilience to suicidal ideation [22].

### 2.3. Procedure

This study was approved by the research ethics committee of the university of one of the authors. Convenience sampling was conducted. Following specialised training about suicide in adolescents and young people, professionals from Educational Guidance Teams, as well as school and high school management staff, were sent a detailed explanation of the study and were asked to collaborate. Those interested in participating, who were the same people who received the training, were emailed an online link to a Google Form. The link was also shared through social media platforms such as Facebook, X (formerly Twitter), and digital communication tools such as WhatsApp and Telegram. Participation in the study was voluntary, and the adolescents had to explicitly express their desire to participate in the study, as well as obtain consent from parents or guardians. To access the tests, the adolescents had to provide an email address for one or both parents or guardians. Parents could request specific information about their children’s results after their participation in the study. During data collection, a high suicidal vulnerability was observed in three participants, so the first author of this study contacted the parents by email and later by videoconference.

### 2.4. Data Analysis

Descriptive analyses were conducted on all variables to determine correlation and significance levels. Subsequently, multiple linear regression was applied to determine which variables predicted high levels of resilience in this sample. Additionally, instrument reliability was measured using the Omega coefficient [37], which is not affected by the number of items or participants [54]. The statistical analyses were performed using G*Power [55,56,57], SPSS version 23.0, and AMOS 23. Statistical significance was considered when *p* < 0.005.

## 3. Results

Descriptive results showed low scores on protective variables and high scores on risk variables in this sample. The reliability of the study sample was assessed using McDonald’s Omega [37] and was adequate on all variables (Table 2).

As anticipated, the protective variables exhibited a positive, significant correlation (*p* < 0.05), while the risk variables exhibited an inverse, significant correlation (Table 3). Notably, social support, and resilience demonstrated the strongest positive correlation (r = 0.98; *p* < 0.01), while entrapment demonstrated a strong correlation with the levels of depression, anxiety, and stress (r = 0.99; *p* < 0.01). Additionally, there was a strong inverse correlation between psychological well-being and depression, anxiety, and stress (r = −0.98; *p* < 0.01).

Preliminary fit data for the proposed models indicated an adequate level of fit [58]. More specifically, the prediction of suicidal vulnerability (SENTIA brief) as an independent variable from the demographic variables and risk factors demonstrated suitable independence of errors through the Durbin–Watson (DW) test (DWsentia = 1.93), as well as absence of multicollinearity through the variance inflation factor (VIF) (VIFsentia = 2.84). Moreover, the two indices of fit for the model predicting the effects of demographic variables and protective factors on the independent variable indicated that resilience (SRSA-18) also demonstrated an adequate fit of the protective model to the data (DWSRSA-18 = 1.97; VIFSRSA-18 = 4.13). The results from the hierarchical multiple regression analysis indicated that some of the sociodemographic and protective and risk variables could predict the level of vulnerability and resilience to suicide (Table 4). On the one hand, Model 1, consisting of a set of sociodemographic and risk variables, was found to be significant and explained 82.8% of suicidal vulnerability in this sample (F(1, 77) = 1131.21; *p* < 0.01). The sociodemographic variables that were most predictive of suicidal vulnerability in self-reported LGBTIQ+ adolescents were not having completed school (β = 1.53; CI (95%) = −1.12–1.99; *p* < 0.01), transgender orientation (β = 2.86; CI (95%) = 2.11–3.46; *p* < 0.01), and having family problems (β = 24.13; CI (95%) = 0.82–0.91; *p* < 0.01). The study found that feeling trapped was the strongest predictor of suicidal vulnerability (β = 6.11 CI (95%) = 1.49–4.27; *p* < 0.01), followed by depression (β = 3.22; CI (95%) = 1.45–2.11; *p* < 0.01) and anxiety (β = 1.76; CI (95%) = 1.65–1.87; *p* < 0.01). On the other hand, Model 2 accounted for 84.5% of the variance in the demographic and protective variables that were most predictive of resilience to suicide (F(1, 77) = 2426.11; *p* < 0.01). The demographic variable that was found to be most predictive was having a homosexual orientation (β = 4.43; CI (95%) = 3.28–4.22; *p* < 0.01). The protective factors associated with a higher level of resilience to suicide attempts included psychosocial well-being (β = 9.14; CI (95%) = 8.01–9.32; *p* < 0.01), high parental affectivity and communication (β = 9. 18; CI (95%) = 8.11–9.29; *p* < 0.01), positive reframing of problems to a lesser extent (β = 6.86; CI (95%) = 5.65–6.71; *p* < 0.01), and adequate emotional regulation (β = 5.67; CI (95%) = 2.69–3.37; *p* < 0.01).

## 4. Discussion

This study sought to determine what risk and protective factors predicted vulnerability or resilience to suicidal behaviour in LGBTIQ+ adolescents who had made previous suicide attempts.

Previous studies have reported that Spanish LGBTQ+ adolescents are more vulnerable to suicide than adolescents who do not belong to sexual minorities [59]. Further analysis of the risk model identified in this study shows that emotional disturbances such as feeling trapped is an important predictor of the risk of new suicide attempts among LGBTQ+ adolescents, more so than anxiety and depression. A person thinking that the adverse circumstances they experience are uncontrollable and inescapable (entrapment) is a concept that forms part of several theories of suicide [60,61], especially related to LGBTIQ+ people [14,62].

Our study, like previous research, also found that the family plays a tremendously important role in the suicidal behaviour of sexually diverse adolescents and young people [63]. Hence, it is essential to offer effective communication strategies to families of sexually diverse adolescents as a procedure to minimise suicidal vulnerability, especially for transgender adolescents [64,65]. Along these lines, the point at which transgender youth may exhibit behaviours that do not conform to their assigned sex is well before the point at which sexual minority youth identify their sexual orientation [66]. Such situations may lead to more intense pressures from their environment to conform to normative tendencies for longer, starting at younger ages, with differential mental health effects and higher risk of subsequent suicide more likely, with more pernicious effects on the transgender subgroup. This could be explained by the fact that the support of one’s most immediate social environment (e.g., family and friends) limits the risk of suicidal behaviour by increasing levels of emotional stability (e.g., seeking help from family and friends at the first thoughts of suicide). Our model of resilience to suicide in this study identified protective factors. In order of importance according to the beta coefficients, they were family relationships characterised by affection and communication; a sense of well-being with attention to satisfaction with life; the ability to re-evaluate stressful situations; and to a lesser extent, emotional regulation skills and a self-defined sexual orientation as homosexual.

In this regard, the model’s inclusion of family relationships based on communication and affection is consistent with protective action against new suicide attempts [59]. Family support is involved in the development of a well-integrated sexual identity and the maintenance of optimal levels of self-esteem, while parent-initiated attempts to change the sexual orientation of LGTBIQ+ young people are associated with strong feelings of entrapment and consequent suicide attempts.

Another factor that may predict protection against suicide in adolescents is life satisfaction [67]. Our protective model also seems to confirm its importance as a protective factor against suicide attempts in LGBTIQ+ adolescents. However, the level of life satisfaction these people achieve may be lower than that identified in the general population and could depend on the adolescent’s sexual orientation. Previous studies [68] identified lower satisfaction in gay/lesbian than heterosexual people, and even lower levels in bisexual people. Including life satisfaction as a predictor of resilience to suicide in sexual minority youth in our study is in line with these findings. Variability in the stigmatisation experienced by sexual minorities by type may underlie the explanation for these differences in well-being. More specifically, previous studies [69] identified that life satisfaction for LGTBIQ+ people is predicted by appropriate integration and externalisation of sexual orientation identity. We believe that the protective role of life satisfaction in the suicide resilience model in our study, together with the involvement of positive family relationships based on affection and communication that encourage an appropriate sexual identity, suggests that life satisfaction may have a modulating role in the relationship between stressors related to social stigmatisation and suicide resilience.

In addition, our study also highlights the important contribution of regulation strategies in suicidal behaviour after a first attempt in LGTBIQ+ adolescents and young people, in line with the suggestions by Siegmann et al. [70] Our study indicates that, the capacity for cognitive reappraisal and various emotional regulation strategies are involved in resilience to suicide. Cognitive reappraisal is an active coping strategy in which the way in which an event is interpreted is altered and can lead to a reduction in the stress response. It is therefore often understood as an adaptive strategy by allowing modification of emotions and behaviours in a stressful situation [38]. Its counterpart is emotional suppression, an inappropriate strategy of emotional regulation which attempts to block the event by not addressing it or coping with it. Limited access to emotion regulation strategies in LGTBIQ+ adolescents is a significant predictor of the frequency and intensity of suicide attempts as an outcome [10,71]. Adolescents who experience distress and lack regulatory strategies may feel helpless and/or hopeless about their ability to feel better (feel trapped), which may lead them to consider a first suicide attempt as a way of escaping distress.

The involvement of sexual identity as homosexual in our model of resilience to suicide is consistent with suggestions by other authors [72,73] that homosexual adolescents—as a subgroup in the LGTBIQ+ population—experience lower levels of discrimination, affirm their identity to a greater extent, and experience greater connectedness to their general social environment. Acceptance by people close to them (friends and family), self-acceptance of belonging to a sexual minority along with greater social and health resources that increase the ability to be resilient to stigmatisation (e.g., pressure from mainstream groups, this is especially true in conservative heteronormative cultures [74]), and the ability to be more aware of their sexuality together with greater visibility of identity than less defined or emerging groups (e.g., intersex, queer, non-binary, gender fluid and pansexual), are all key protective factors in LGTBIQ+ adolescents that should be considered in any suicide prevention intervention with this group.

Finally, the limitations of this study are related to the difficulties accessing samples of LGTBIQ+ adolescents, which is even more challenging if they have made previous suicide attempts. This aspect is reflected in the size of the sample, which could imply methodological problems that we have tried to control for. Moreover, the study sought to address a specific group using an approach based not only on risk factors, but also on protective factors. We hope that this aspect will encourage other researchers to provide more data on this population, which is underrepresented in most studies on suicide attempts.

## Figures and Tables

**Table 1:** Demographic data of the sample.

	Total (%)	χ^2^	d.f.	Phi
Self-reported sexual orientation		11.31 *	3	0.71
Homosexual (gay/lesbian)	43 (55.1)			
Bisexual	14 (17.9)			
Transgender	13 (16.7)			
Intersex/Queer	8 (10.3)			
Self-reported socio-economic status		1.11 ^ns^	2	0.69
Low	24 (30.8)			
Medium	31 (39.7)			
High	23 (29.5)			
Level of completed education		1.06 ^ns^	2	0.72
None	13 (16.7)			
Primary Education	39 (50.0)			
VET/HNC/High school diploma	26 (33.3)			
Stressful situation (last year)		67.28 **	1	0.93
Yes	69 (88.5)			
No	9 (11.5)			
Triggering situation		1.37 ^ns^	3	0.73
Bullying	38 (48.7)			
Couple Problems	13 (16.7)			
Family problems	16 (20.5)			
Problems with friends	11 (14.1)			
Previous suicide attempts (last year)		6.34 *	1	0.83
Only one	59 (75.6)			
Two or more	29 (24.4)			
ωASQ	0.97			
Total	78 (100)			

VET = Vocational Education and Training; HNC = Certificate of Higher Education; ωASQ = Mcdonald’s Omega [37] was performed for Item 4 of the short scale Ask Suicide-Screening Questions-ASQ from [35] Horowitz et al. (2012); χ^2^ = Chi-square * = *p* < 0.05; ** = *p* < 0.01; ns = no significant; d.f. = degrees of freedom; Phi = effect size.

**Table 2:** Descriptive results of the study variables.

	M (SD)	Min–Max	Skewness	Kurtosis	ω
Emotional Regulation	21.5 (3.4)	10–70	0.26	0.47	0.89
Reappraisal	14.2 (4.7)	9–45	0.51	−0.74	0.91
Social Support	11.6 (1.34)	0–30	0.89	1.13	0.94
Psychological Well-being	16.1 (3.2)	10–50	−1.21	0.68	0.79
Parental Style	9.3 (11.2)	8–48	0.39	−0.43	0.92
Entrapment	15.9 (2.6)	0–16	−0.66	0.77	0.96
Depression, Anxiety, and Stress	57.8 (4.1)	0–63	−0.32	0.53	0.91
Suicidal Vulnerability	3.7 (1.2)	0–4	0.21	0.53	0.98
Resilience to Suicide Attempts	19.2 (7.2)	0–72	0.44	−0.21	0.97

M = Mean; SD = Standard Deviation; Emotional Regulation = Emotion Regulation Questionnaire-ERQ; Reappraisal = The Reappraisal Index (RI); Social Support = The Lubben Social Network Scale-6 (LSNS-6); Psychological Well-being = Psychological Well-being Scale (PWS); Parental Style = Scale for Evaluating Parental Educational Style Towards Adolescents-SEPES; Entrapment = The Entrapment Scale Short-Form (E-SF); Depression, Anxiety, and Stress = The Depression, Anxiety and Stress Scale (DASS-21); Suicidal Vulnerability = The Suicidal Behaviour Assessment Scale (SENTIA brief); Resilience to Suicide Attempts = The Resilience Scale for Suicide Attempts (SRSA-18 Adolescents and Youth); ω = Reliability.

**Table 3:** Correlation and level of significance between the variables.

	ERQ	RI	LSNS-6	PWS	SEPES	E-SF	DASS-21	SENTIA	SRSA-18
ERQ	1	0.54 *	0.68 **	0.91 *	0.71 *	−0.84 **	−0.73 **	−0.71 *	0.92 **
RI	0.54 *	1	0.83 **	0.89 *	0.63 *	−0.91 *	−0.89 **	−0.65 *	0.96 **
LSNS-6	0.68 **	0.83 **	1	0.75 *	0.91 **	−0.83 *	−0.93 **	−0.78 **	0.98 **
PWS	0.91 *	0.89 *	0.75 *	1	0.86 **	−0.89 **	−0.98 **	−0.93 **	0.87 **
SEPES	0.71 *	0.63 *	0.91 **	0.86 **	1	−0.86 **	−0.91 **	−0.84 *	0.85 **
E-SF	−0.84 **	−0.91 *	−0.83 *	−0.89 **	−0.86 **	1	0.89 **	0.99 **	−0.93 **
DASS-21	−0.73 **	−0.89 **	−0.93 **	−0.98 **	−0.91 **	0.89 **	1	0.93 **	−0.95 **
SENTIA	−0.71 *	−0.65 *	−0.78 **	−0.93 **	−0.84 *	0.99 **	0.93 **	1	−0.83 **
SRSA-18	0.92 **	0.96 **	0.98 **	0.87 **	0.85 **	−0.93 **	−0.95 **	−0.83 **	1

ERQ = Emotion Regulation Questionnaire; RI = The Reappraisal Index; LSNS-6 = The Lubben Social Network Scale-6; PWS = Psychological Well-being Scale; SEPES = Scale for Evaluating Parental Educational Style Towards Adolescents; E-SF = The Entrapment Scale Short-Form; DASS-21 = The Depression, Anxiety and Stress Scale; SENTIA brief = The Suicidal Behaviour Assessment Scale; SRSA-18 = The Resilience Scale for Suicide Attempts (Adolescents and Youth); * = Significance *p* < 0.05 (bilateral); ** = Significance *p* < 0.01 (bilateral).

**Table 4:** Predictive models of vulnerability and resilience to suicide with demographic and psychosocial risk and protection variables.

Models and Variables	R^2adj^	SE	*F*	*t*	β	CI (95%) (β)		*φ* ^2^
						L.L.	U.L.	
*M*_1_	0.828	2.44	1131.21 **	45.11 *				71.3
Age					0.35	−0.21	1.61	
Level of education (none)					1.53	−1.12	1.99	
Self-reported sexual orientation (transgender)					2.86	2.11	3.46	
Triggering situation (family problems)					4.13	0.82	0.91	
Depression					3.22	1.45	2.11	
Anxiety					1.76	1.65	1.87	
Entrapment					6.11	1.49	4.27	
*M*_2_	0.845	2.86	2426.11 **	71.11 **				83.8
Self-reported sexual orientation (homosexual)					4.43	3.28	4.22	
Emotion Regulation					5.67	2.69	3.37	
Well-being					9.14	8.01	9.32	
Reappraisal (reframing)					6.86	5.65	6.71	
Affection and communication					9.18	8.11	9.29	

M1 = Risk prediction (SENTIA brief); M2 = Prediction of protection (SRSA-18); R^2adj^ = Adjusted R-squared; SE = standard error; F = test statistic (ANOVA); * *p* < 0.05, ** *p* < 0.01; ns = non-significant; t = predictive variable test statistic; β = result of regression or beta equation; CI (95%) = confidence intervals; LL = lower limit; UL = upper limit; *φ*^2^ = effect size.

## Data Availability

The data presented in this study are available on request from the corresponding author. The data are not publicly available due to privacy or ethical restrictions.
